# Editorial: Towards equitable health systems for universal health coverage (UHC) in sub-Saharan Africa

**DOI:** 10.3389/frhs.2023.1217844

**Published:** 2023-05-30

**Authors:** John E. Ataguba, Eugenia Amporfu, Daniel M. Achala, Juliet Nabyonga-Orem

**Affiliations:** ^1^ Department of Community Health Sciences, Max Rady College of Medicine, Rady Faculty of Health Sciences, University of Manitoba, Winnipeg, MB, Canada; ^2^ African Health Economics and Policy Association, Accra, Ghana; ^3^ Health Economics Unit, School of Public Health, Health Sciences Faculty, University of Cape Town, Cape Town, South Africa; ^4^ Department of Economics, Kwame Nkrumah University of Science and Technology, Kumasi, Ghana; ^5^ Universal Health Coverage and Life Course Cluster, World Health Organization, Brazzaville, Congo; ^6^ Centre for Health Professions Education, Faculty of Health Sciences, North-West University, Potchefstroom, South Africa

**Keywords:** universal health coverage, sub-Saharan Africa, social determinants in health, financial protection, health service coverage

**Editorial on the Research Topic** Towards equitable health systems for universal health coverage (UHC) in sub-Saharan Africa

The global commitment to universal health coverage (UHC) goals is evident in many countries' health sector strategies and UHC Roadmaps. UHC requires that everyone has access to the needed health services of sufficient quality to be effective, without facing any financial hardships due to the need to use such services (1). UHC underpins the Sustainable Development Goal (SDG) 3 “Good health and well-being”. There are two major dimensions of UHC that countries are encouraged to assess and monitor; financial protection and coverage of quality health services (2, 3). Financial protection is about ensuring that health service use does not affect a household's capacity to purchase necessities like food, shelter, clothing, etc. Coverage with quality health services, on the other hand, is about using needed health services of sufficient quality to be effective without any barriers. As we move towards the 2030 SDG timeline, of concern is the slow progress in Africa as many African households face significant financial barriers to accessing health services, including impoverishment and financial catastrophe (4), and coverage with essential health services remains low.

The UHC average Service Coverage Index (SCI) for the World Health Organization (WHO) African Region increased by only 22 index points between 2000 (24%) and 2019 (46%), while the population spending more than 10% of household expenditure on health services, an indication of financial catastrophe, increased from 51 to 87 million (5). These regional averages mask the persistent inequalities in service coverage and financial risk protection. Using 2021 data, the World Health Organization estimates that most countries in sub-Saharan Africa (Figure 1) have a UHC service coverage index for essential health services lower than 50, where 100 represents full-service coverage. While the UHC service coverage index does not explicitly account for the quality of health services, it exceeded 64 in only Mauritius (66), South Africa (71), Cabo Verde (71), and Seychelles (75). Somalia (27) and Chad (29) have the worst UHC coverage levels in sub-Saharan Africa (Figure 1). Although attaining UHC requires more than a well-functioning health system, health systems barriers still play a critical role (6).

**Figure 1:**
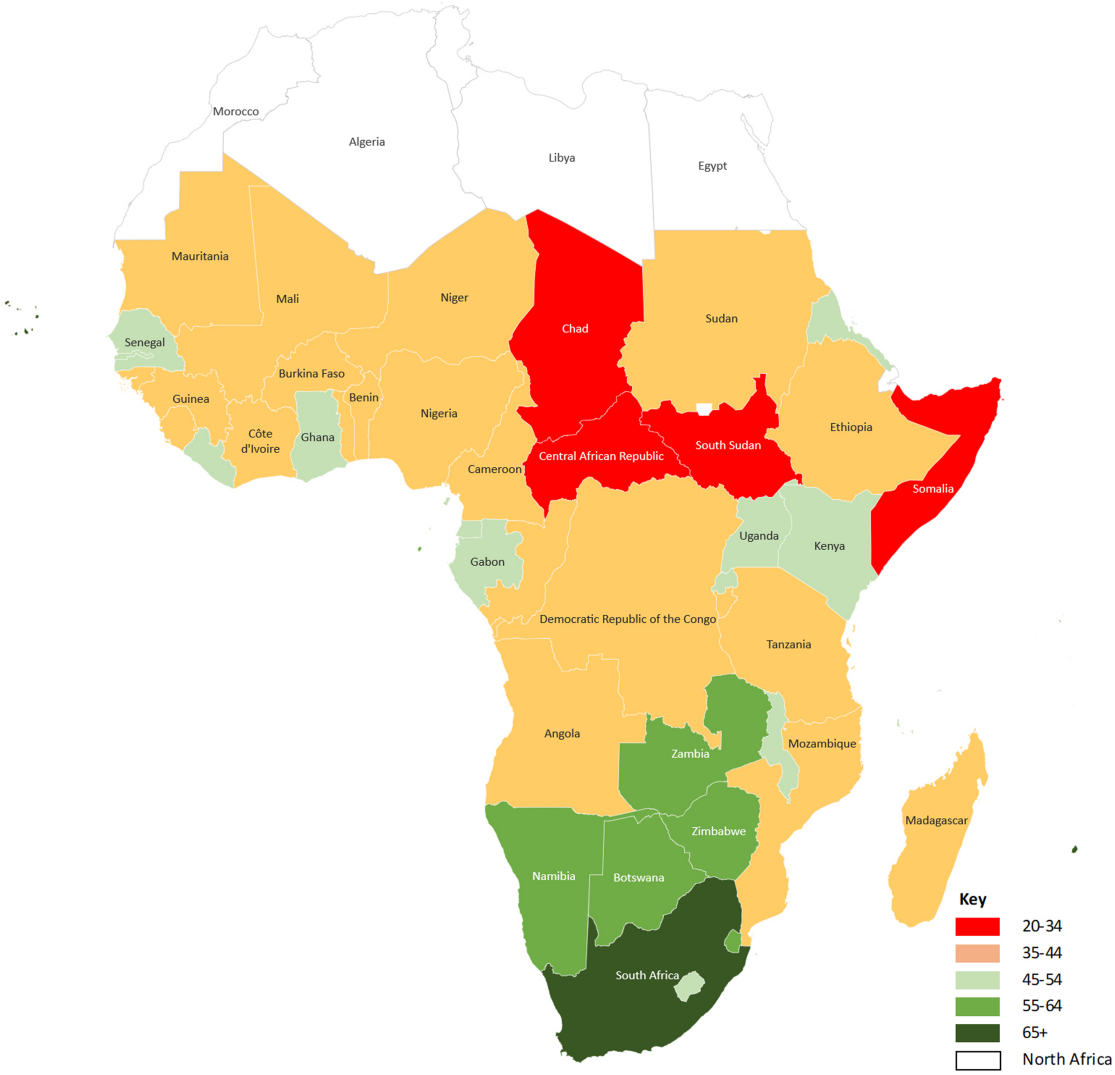
UHC service coverage index in sub-Saharan Africa, 2021. World Health Organization, Global Health Observatory Data Repository (https://www.who.int/data/gho). UHC service coverage index ranges between 0 and 100, with 100 implying perfect service coverage.

In its 2013 World Health Report, the WHO calls for intensifying UHC research to explore solutions to tackle inequities and close the gaps between population groups to leave no one behind (3). This call is even more relevant as we draw closer to 2030. There are no simple solutions to reaching unreached populations, and to address this gap, several countries have turned to pilot projects to explore what works. Positive results have been recorded in several cases, but scaling these to national levels is challenging. The challenge lies in a limited understanding of what explains observed results, as evaluation studies often fall short of exploring underlying mechanisms.

This Research Topic published an initial collection of six research papers covering different countries to respond to the call for research and the need to provide policy-relevant evidence to assist countries in fast-tracking progress towards UHC (Aregbeshola and Olaniyan, Chauluka et al. Mchenga et al. Oladimeji and Fatusi, Oyando et al.) (7).

Employing realist approaches Oladimeji and Fatusi elaborate a middle-range theory highlighting enabling and constraining factors that explained the results of the safe motherhood initiative in Ondo State—Nigeria. Notably, equipping health facilities with health supplies, providing free services, and matching pregnant women with community extension workers, led to positive results. While this was a study from the Ondo State in Nigeria, the methodological rigor engenders a learning experience with wider application in other low-income countries.

Chauluka et al. noting the minimal health insurance coverage for women in Malawi, found that education, occupation and wealth are significant social determinants of health insurance coverage among women in the country. Prioritising these social determinants of health insurance enrolment is critical for increasing insurance coverage for women in Malawi.

Oyando et al. highlight the gaps in aligning service provision to the need for health services in their study to assess horizontal equity in screening and treating hypertension, a significant public health challenge in Kenya. They further highlight the importance of critical social determinants, including sex, body mass index, wealth, area of residence, and employment status, in explaining observed inequities in screening and treating hypertension in Kenya.

Mchenga et al. developed a UHC index for Malawi using indicators for UHC's two broad dimensions; service coverage and financial protection. Drawing from many data sources, the authors estimated Malawi's UHC index at 69.7%. Service coverage was estimated at 51.6%, which lies in the range indicated in Figure 1. The authors highlight the need for policies to address the two UHC dimensions and reduce group inequalities.

In Nigeria, where the maternal mortality ratio remains high, Aregbeshola and Olaniyan assessed horizontal inequity using maternal and reproductive health services. Although there was relative equity in meeting the need for family planning among women in Nigeria, they found significant inequity in women's use of postnatal care, caesarean section delivery, and modern contraceptive to the advantage of wealthier groups. The authors highlight the importance of addressing the needs of women in achieving the SDGs. In their systematic review paper in the Reproductive Health journal, (7) examine factors influencing access and use of youth-friendly sexual and reproductive health services (YFSRHS) in sub-Saharan Africa. They identified both structural (e.g., unfavourable attitudes of health service providers) and individual-level barriers (e.g., limited knowledge and information among the youths) to utilising YFSRHS in sub-Saharan Africa. Importantly, community outreaches and health education emerged as critical factors facilitating the use of YFSRHS.

## Conclusion

Sub-Saharan Africa's UHC research agenda is vast, as many of the region's countries have low UHC service coverage index values. As demonstrated by the research summarised in this editorial, significant inequities exist in many health systems. Individuals still face substantial access barriers, especially women, the youth and those from disadvantaged backgrounds. The research papers also demonstrate the poor alignment of service provision to need and the importance of the social determinants as drivers of health inequalities. This accentuates the need for multisectoral action by governments to address the challenges of accessing health services, close the gaps between groups, especially the rich and the poor, and leave no one behind in the move towards UHC. While policy action in other relevant sectors is crucial, within the health sector, paying attention to tailored and contextual approaches that respond to the health needs of the different groups, ensuring that people are aware of their entitlements where such exist, and mitigating financial, physical and other access barriers to ensure people can access quality health services is critical. For equity and to achieve UHC, it is essential to ensure that people finance health services according to their ability to pay but utilise health services based on need without any barriers.
